# Discrimination of Transgenic Rice containing the Cry1Ab Protein using Terahertz Spectroscopy and Chemometrics

**DOI:** 10.1038/srep11115

**Published:** 2015-07-08

**Authors:** Wendao Xu, Lijuan Xie, Zunzhong Ye, Weilu Gao, Yang Yao, Min Chen, Jianyuan Qin, Yibin Ying

**Affiliations:** 1 College of Biosystems Engineering and Food Science, Zhejiang University, 866 Yuhangtang Rd., 310058 Hangzhou, PR China; 2 Department of Electrical and Computer Engineering, Rice University, Houston, TX 77005, USA

## Abstract

Spectroscopic techniques combined with chemometrics methods have proven to be effective tools for the discrimination of objects with similar properties. In this work, terahertz time-domain spectroscopy (THz-TDS) combined with discriminate analysis (DA) and principal component analysis (PCA) with derivative pretreatments was performed to differentiate transgenic rice (Hua Hui 1, containing the Cry1Ab protein) from its parent (Ming Hui 63). Both rice samples and the Cry1Ab protein were ground and pressed into pellets for terahertz (THz) measurements. The resulting time-domain spectra were transformed into frequency-domain spectra, and then, the transmittances of the rice and Cry1Ab protein were calculated. By applying the first derivative of the THz spectra in conjunction with the DA model, the discrimination of transgenic from non-transgenic rice was possible with accuracies up to 89.4% and 85.0% for the calibration set and validation set, respectively. The results indicated that THz spectroscopic techniques and chemometrics methods could be new feasible ways to differentiate transgenic rice.

Rice is a very important part of the worldwide agricultural crop family. However, rice producers suffer large economic losses due to the damage caused by insects. Although the utilization of chemical pesticides can alleviate the damage to some extent, it increases the cost of production and leaves pesticide residues. Planting insect-resistant transgenic plants may be an optional solution. The first transgenic plant was formed in 1993 by inserting the *Bacillus thuringiensis* (Bt) gene. This transgenic plant could express the Cry1Ab protein, which protects against pests^1^. Although genetically modified organisms (GMOs) contribute to rice production to a certain extent, the influences of GMOs have not been completely investigated. Controversies over GMOs occur because of their potential negative effects on human beings and other non-target creatures, such as butterflies, wasps and bees^2^,^3^. Therefore, the production and marketing of Bt crop products are strictly regulated by the European Union, as well as many other countries and regions^4^,^5^. Therefore, affordable and effective methods for rapid identification of GMOs are of crucial importance.

A variety of methods, including the polymerase chain reaction (PCR)^6^, enzyme-linked immune sorbent assay (ELISA)^7^, biosensors^8^, microarrays^9^, chips^10^, electrophoresis^11^ and mass spectrometry^12^, have been proven effective in detecting GMOs. However, their shortcomings, such as high costs, large time-consumption and difficult operations, cannot be ignored^13^. As non-destructive tools, spectroscopic techniques are fast and easy to operate without complicated sample preparations. Near infrared (NIR), visible near infrared (VIS-NIR) and Raman spectroscopic techniques combined with chemometrics methods have shown their success in the rapid identification of GMOs^14^,^15^,^16^,^17^,^18^. Although many of the spectroscopic techniques mentioned above have been used to identify GMOs, little attention has been paid to the use of terahertz (THz) spectroscopy for the detection of GMOs. In 2014, Liu *et al.*^19^ showed that THz spectroscopy combined with a learning affinity propagation clustering algorithm (ALAP) and support vector machine (SVM) could be utilized for the identification of different transgenic cottons with accuracy of up to 97.794%.

The THz region of the electromagnetic spectrum, lying between millimeter radio waves and far infrared light waves (from 0.1 THz to 10 THz), exhibits properties of both sides of the electromagnetic spectrum^20^,^21^,^22^. The absorption of THz waves by molecular and biomolecular systems are dominated by the excitation of intramolecular and intermolecular vibrations^23^. Due to its potential applications, such as security detection^22^,^24^, medical science detection^25^, biological detection^26^,^27^ and agricultural detection^23^, THz spectroscopy has become one of the most dynamic fields of scientific research. However, until now, there have been no studies focusing on transgenic food detection using terahertz spectroscopy with chemometrics methods.

In this work, transgenic rice (Hua Hui 1), which contains the Cry1Ab protein, its parent plant (Ming Hui 63) and the Cry1Ab protein were selected as subjects for terahertz time-domain spectroscopy (THz-TDS) identification of GMOs. Chemometrics methods, such as principal component analysis^28^ (PCA), discriminate analysis (DA)^17^ and partial least squares (PLS) regression^29^ in conjunction with derivative pretreatments, were used to test the performance of THz-TDS in the detection of GMOs.

## Results

### Spectra analysis

**Figure 1:**
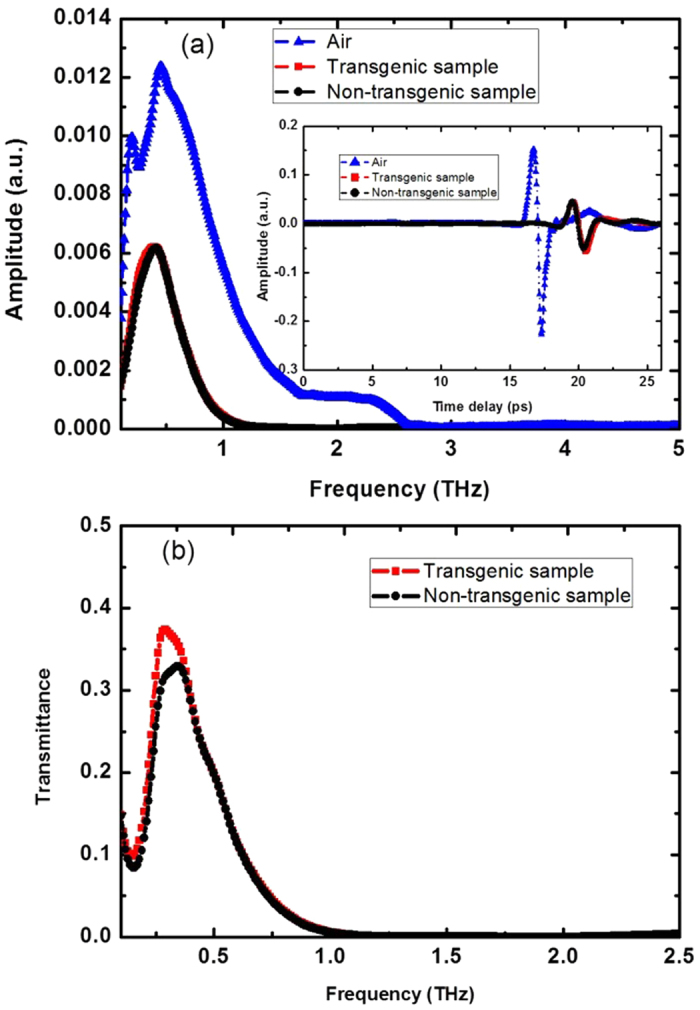
Transmitted THz amplitude spectra, obtained via Fourier transform of time-domain signals. (**a**) Transmitted THz amplitude of transgenic rice, non-transgenic rice and air. The inset shows their time-domain signals; (**b**) average transmittance of transgenic samples and non-transgenic samples.

Time-domain spectra of the Cry1Ab protein and rice samples were collected with three replicates and each of them was averaged. Figure 1 presents the transmitted THz amplitude spectra, obtained through Fourier transform of the time-domain signals.

The inset of Fig. 1a shows the average amplitudes of transgenic rice (red square) and non-transgenic rice (black circle) in time domain. We could find that the averages of the amplitudes of transgenic rice and non-transgenic rice overlapped and were hard to differ from each other. Compared with the average amplitude of air (reference), the average amplitudes of transgenic rice and non-transgenic rice are much smaller. This result is due to the rice pellet’s absorbance and refraction, and is also reflected in the frequency-domain region, indicating that the frequency-domain amplitude of the rice samples is much smaller than that of air. High-frequency THz waves are strongly absorbed by the rice samples, and the amplitude decreases to ~0 above 1.2 THz as shown in Fig. 1a.

Two average transmittance curves of rice according to their genotype are shown in Fig. 1b. The transmittance curves are lower than 40% and overlap above 0.5 THz. We can see that the average transmittance of the transgenic samples is higher than the non-transgenic samples in the region from 0.2 to 0.4 THz, with the most obvious difference at 0.4 THz. The peaks from 0.2 to 0.4 THz may be caused by the rice starch because the starches from different agro-products show different properties in the THz region^30^. However, the spectra of the transgenic and non-transgenic rice varied (data not shown), and the average transmittances of the transgenic ones are not always higher than those of their parents from 0.2 to 0.4 THz. That is, it is difficult to discriminate transgenic samples from non-transgenic samples based on their frequency-domain curves only. Chemometrics methods can highlight the chemical differences between samples and reduce the variation due to physical effects. Therefore, chemometrics methods were used to build a qualitative model for transgenic rice and its parent’s discrimination.

**Figure 2:**
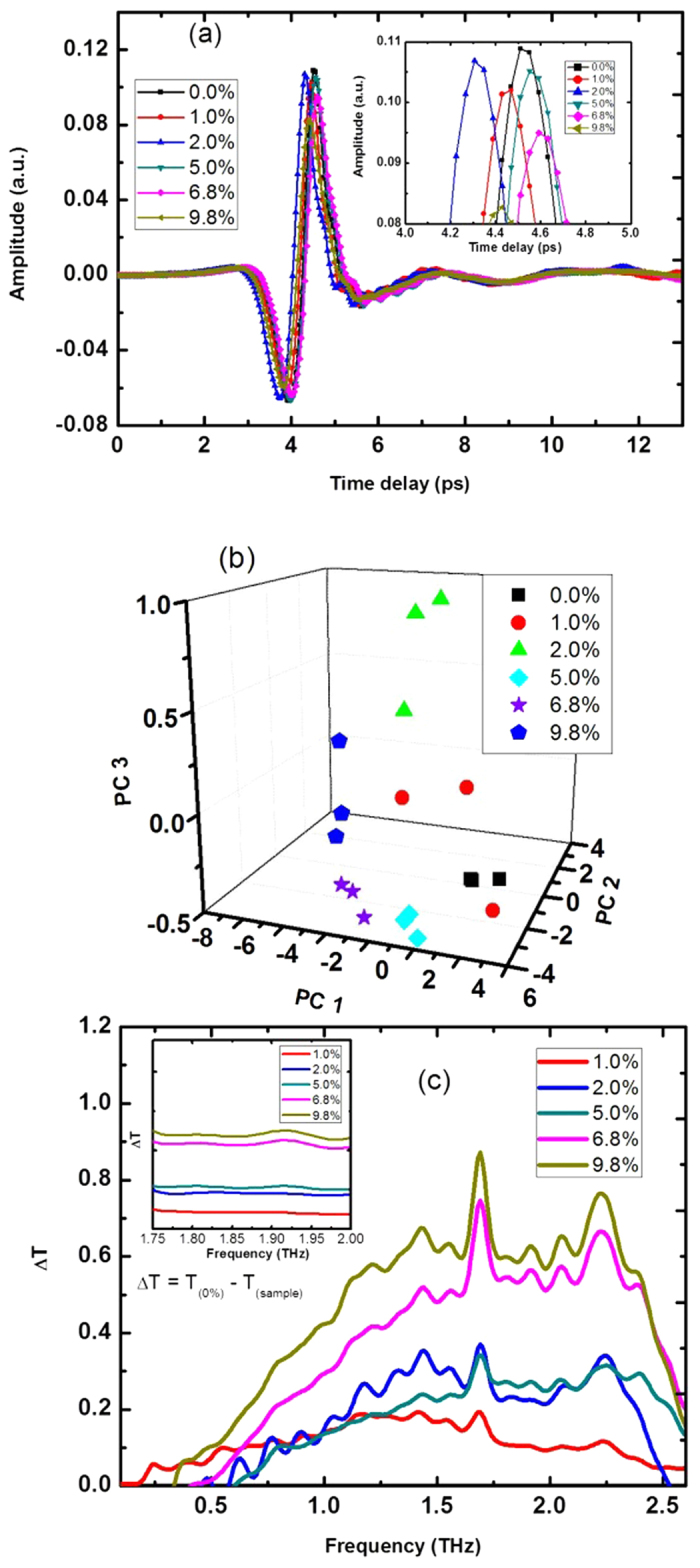
Results from the Cry1Ab pellets with different concentrations. (**a**) THz time-domain waveforms of the Cry1Ab pellets with different concentrations; (**b**) three-dimensional score plot of the first three principle components for the Cry1Ab pellets with different concentrations; (**c**) the difference transmittance of the Cry1Ab pellets with different concentrations.

Since transgenic rice contains Cry1Ab protein, we conducted the experiment for pellets of Cry1Ab protein and PE with different mixing ratios. The plots of the time-domain spectra, principle scores and difference transmittance of the Cry1Ab protein pellets are shown in Fig. 2. Figure 2a shows that there is no significant time delay between the time-domain spectra of PE (with a 0.0% concentration of the Cry1Ab protein) and the Cry1Ab pellets. However, the peak amplitude of the average spectrum of the PE pellet is higher than that of the Cry1Ab pellet. The peak amplitude generally decreases as the concentration level increases. However, the peak amplitude of Cry1Ab pellet with a 1% concentration is lower than that of the Cry1Ab pellets with 2% and 5% concentrations. This result may be due to the non-uniform distribution of the Cry1Ab protein in the Cry1Ab protein pellet and the PE complex.

The three-dimensional (3D) principal component score plot of the transmittance spectra of the Cry1Ab pellets is shown in Fig. 2b. The first three principal components (PCs) contain the most spectral variations 97.870% (93.396%, 2.654%, 1.821% for PC1, PC2, PC3, respectively). Based on this figure, the samples are clearly divided into six different groups according to their Cry1Ab contents. The PC1 value for the PE pellets is the biggest. In addition, the PC1 value generally decreases with an increase in the Cry1Ab protein content. Figure 2b shows the discriminating ability of PCA and indicates that transmittances of the Cry1Ab pellets could be differentiated based on the concentration of the Cry1Ab protein. This agrees with Xie’s result^17^.

**Figure 3:**
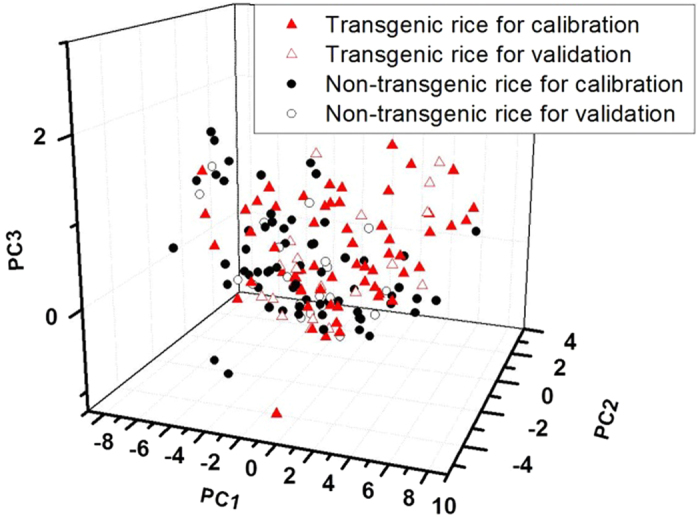
Three-dimensional score plot of the first three principle components for the transgenic and non-transgenic rice samples.

To increase the difference between the Cry1Ab pellet samples, the difference transmittance method is used and the results are shown in Fig. 2c. The difference transmittance is defined as


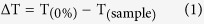


where T_(0%)_ is the transmittance of the PE pellet and T_(sample)_ is the transmittance of a Cry1Ab pellet sample. Strong peaks at 1.20, 1.40, 1.70, 2.05 and 2.20 THz appeared, as shown in the difference transmittance spectra in Fig. 3c. Because L-glutamic acid shows absorption peaks at 1.20 and 2.05 THz, L-cysteine shows absorption peaks at 1.40 and 1.70 THz, and L-histidine shows an absorption peak at 2.20 THz^27^, these peaks may be assigned to amino acids. Because the Cry1Ab protein is mainly composed of different types of amino acids, these characteristic peaks of the Cry1Ab protein may be caused by its amino acids. These spectra overlapped each other, and no clear correlation between the difference transmittance and the concentration was shown, except for the 1.30–1.60 THz and 1.75–2.00 THz bands. The inset of Fig. 2c shows the transmittance spectra of the Cry1Ab pellet samples in the range of 1.75–2.00 THz. In the region of 1.75–2.00 THz, the waveforms of the difference transmittance spectra could be distinguished from each other, and the amplitude increased with the concentration of the Cry1Ab protein. That is, the difference transmittance shape changed with the Cry1Ab protein concentration and the PE mixing ratio and could be separated in the 1.30–1.60 THz and 1.75–2.00 THz bands.

### PLS analysis of Cry1Ab pellets

Multiple regression methods, such as PLS, have been successfully applied to the quantitative analysis of THz spectra and allow for the correlation of the THz spectra with the concentration^31^,^32^. In our study, 0.1–2.6 THz was chosen to build the calibration models using the PLS algorithms and validated by cross-validation. The correlation coefficients (*r*) of the calibration and cross validation (*r*_*c*_ and *r*_*cv*_), the root-mean-squared errors of the calibration set (RMSEC) and the root-mean-squared errors of the cross validation (RMSECV) were used to assess the models. First and second derivative methods were the pretreatment methods. The results are shown in Table 1. Table 1 shows that the correlation coefficient *r*_*c*_ of the raw spectra is 0.9696. The use of the derivative process could remove an additive baseline, correcting for baseline shifts^18^. Using derivative pretreatment could decrease the noise and may produce a better result. This table demonstrates that the best model with high *r*_*c*_ (0.9725) and *r*_*cv*_ (0.8926) is obtained from spectra using the first derivative pretreatment. When using spectra pretreated with the second derivative method, the correlation coefficients are good with *r*_*c*_ equal to 0.9715 and *r*_*cv*_ equal to 0.8912. Considering that a good model should have low RMSEC and RMSECV values^33^, the modeling result using the first derivative obtained the lowest RMSEC (0.83%) and RMSECV (1.61%) values and is better than the other two algorithms. The result showed that different concentration of Cry1Ab protein could be differed from each other with the lowest concentration reaching to 1%. However, the amount of Cry1Ab protein in transgenic rice is much less than 1%. In our study, chemometrics methods were used to highlight the chemical differences between transgenic rice and its parent.

### Spectral range selection

All of the transmittance spectra of the rice samples were analyzed using the DA method (linear discriminant analysis). To obtain the best spectral range for calibration, spectrum range selection is needed. Because the frequency amplitudes of the rice samples were ~0 above 1.2 THz, the waveband above 1.2 THz was not chosen to build a model. In this study, the discrimination accuracies of the DA model were calculated from the spectral range starting at 0.1 THz and ending between 0.2 and 1.2 THz with an interval of 0.1 THz because the transmittances of the samples decrease to 0 above 1.2 THz. All of the spectra were analyzed without any data pretreatments. The results are shown in Table 2.

Table 2 indicates the highest discrimination accuracy of 85.3% in the regions of 0.1–0.6 THz, 0.1–1.0 THz, 0.1–1.1 THz and 0.1–1.2 THz. In Fig. 1b, the transmitted THz amplitude spectra of the transgenic and non-transgenic samples overlap above 0.6 THz. Because the analysis based on the region from 0.1 to 0.6 THz shows the best accuracy with the least spectra data, it is selected for the DA analysis. Compared with the best accuracy in Table 2 (85.3%), PCR^6^, ELISA^7^, biosensor^8^, electrophoresis^11^ and mass spectrometry^12^ methods could achieve better detection results. However, they generally need high cost and professional operators. Moreover, spectroscopic techniques are fast and easy to operate. Therefore, we built calibration and validation models using terahertz spectroscopy to search for a fast and easy method and hope that it can be used for large numbers of samples.

PCA was applied to all of the transgenic and non-transgenic sample spectra to determine more about the discrimination trend. According to the results of the range selection (Table 2), only the range from 0.1 to 0.6 THz was used for analysis. Figure 3 shows the 3D principal component score plot of the first three score vectors. The initial three factors, which were derived from the first derivative spectra and account for most (97.561%) of the spectral variations (94.055%, 2.535%, and 0.971% for the first three principal components, PC1, PC2, and PC3, respectively), were used to make the differences more clear. Based on this figure, the samples cannot be clearly divided into two groups due to the overlap of some samples. Figure 3 also shows that the transgenic samples and their parents have positive PC2 scores with few exceptions (three non-transgenic samples and one transgenic sample). Compared with the non-transgenic samples, more transgenic samples had positive PC 1 and PC 3 scores. However, it is hard to find significant clustering trends in Fig. 3. PCA fits a subspace with respect to the optimized maximum variance of the data structure^17^. Therefore, discriminant analysis was applied to improve the separation of the groups.

### DA analysis

DA was performed on the first 10 PCs because the first 10 PCs contain more than 99.5% of the variation in the raw data. Table 3 shows the results of the DA model using the raw THz spectra and the spectra with different pretreatments (first derivative and second derivative).

The results indicate that THz spectroscopy shows good potential for the discrimination of rice with different genotypes in the region from 0.1 to 0.6 THz. The accuracies of the DA calibration model and validation model reach 85.4% and 85.0%, respectively. Compared with the discrimination result from the model using the raw spectra, the one using the spectra with the first derivative pretreatment achieves a better discrimination accuracy than the calibration set. However, the discrimination accuracy of the validation set is the same as that of the raw spectra. The model using the spectra with the second derivative pretreatment produces better results than that using the raw spectra, and 20 samples are misclassified; for raw spectra, 24 samples are misclassified. The results from the derivative spectrum achieved better accuracies than that of the raw spectrum, which agrees with Xie’s result^17^. This may due to the derivative process, which increases the signal.

**Figure 4:**
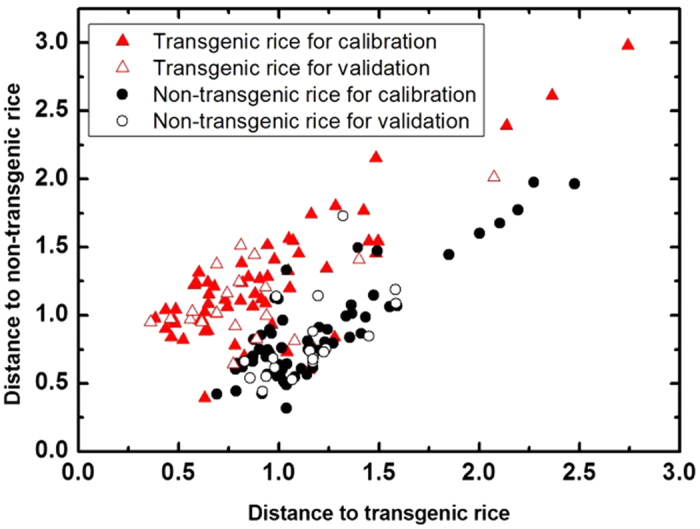
Discrimination results for the transgenic and non-transgenic rice samples using the DA method and the THz spectra with the first derivate pretreatment.

Figure 4 shows a plot of the Mahalanobis distance of the THz spectroscopy of the rice samples with the first derivative pretreatment. This plot shows transgenic rice and non-transgenic rice divided into two groups with a few samples overlapping each other. Therefore, the DA method demonstrates good capability for differentiating the rice with different genotypes.

By applying derivative pretreatments to THz spectroscopy in conjunction with the DA model, identifying transgenic and non-transgenic rice is available with accuracies up to 89.4% and 85.0% for the calibration and validation models, respectively. On the other hand, we build a discriminate model using partial least squares discriminant analysis (PLSDA) method (an application based on PLS method), finding that PLSDA could not discriminate transgenic rice and its parent well with the best discriminate accuracy only reach to 65.0% (data not shown). This may be because the calculation of Mahalanobis distance was more suitable for discriminating transgenic rice and its parent.

## Discussion

This study focuses on classifying transgenic and non-transgenic rice based on THz spectroscopy combined with the PCA and DA models and derivative pretreatments. THz spectroscopy is a relatively powerful tool for differentiating transgenic rice and their parents with accuracies reaching 89.4% and 85.0% for the calibration and validation models, respectively. Spectroscopic techniques offer the benefits of avoiding time-consuming recalibration work for each sample and costly chemical and sensory analyses. Determining transgenic samples using spectroscopic techniques is valuable, and this study shows the potential of THz spectroscopy for transgenic agro-products discrimination. Further studies are needed to build more valuable and robust models to discriminate other rice varieties.

## Methods

### Samples

Two sets of rice, transgenic samples (Hua Hui 1) and its parental line (Ming Hui 63), were grown in separate fields under the same climate conditions and fertility and were harvested at the same time. Hua Hui 1 has a certificate of security with the transgenic Bt gene. The storage conditions are the same in Ref. [18]. The concentration of transgenic protein is less than 2.5 μg g^−1^ in transgenic rice and is too low to be detected in its parent^34^,^35^. Each of the rice samples was shelled, ground into a powder (DFT-50, Dade, Zhejiang, China), meshed (100 mesh sieve) and dried overnight (50 °C). Then, the powder was pressed into pellets under 15 to 20 MPa. Only the pellets with smooth surfaces and without any cracks were suitable for measurement. Each pellet weighted approximately 200 mg and was 1.2 ± 0.1 mm thick and 13 mm in diameter. A total of 163 samples (81 Hua Hui 1 and 82 Ming Hui 63) with different ripeness and plumpness degrees were measured by THz-TDS (room temperature 23 ± 1 °C, less than 1% relatively humidity in a nitrogen atmosphere). All of the samples were measured three times.

The Cry1Ab protein (salt free, M. P. Carey CWRU, Cleveland, Ohio, USA) was uniformly mixed with dried polyethylene (PE, Sigma-Aldrich, St. Louis, MO.) and pressed into a pellet under a pressure of 15-20 MPa and measured using THz-TDS. A total of 18 samples with six different Cry1Ab concentrations (0, 1%, 2%, 5%, 6.8%, and 9.8%) were measured using THz-TDS. Each pellet was measured three times repeatedly with thicknesses approximately 1 mm.

Approximately 3/4 of the rice samples were randomly selected to build a calibration set, and the other 1/4 were used to build the validation set in this work. Therefore, there were 123 samples in the calibration set and 40 samples in the validation set, respectively.

### Equipment

THz time-domain spectra of all of the samples, including rice, Cry1Ab protein and PE pellets, were collected using by a Z-3 terahertz time-domain spectrometer system (Zomega Corporation, East Greenbush, NY, USA) with a low-temperature (LT) GaAs photoconductive antenna as the THz emitter and a ZnTe electro-optical crystal as the THz detector. The spectral range was 0.1–2.6 THz with a frequency resolution of 10 GHz and a signal noise ratio (SNR) of more than 3000. The humidity is less than 1% after nitrogen purging.

The sample pellets were measured using THz transmittance mode. Before sample collection, a spectrum of air was collected as the reference spectrum. All of the spectra were collected after the spectroscopy system warmed up for half an hour to reach a stable state.

### Spectral Data Pretreatment and Chemometrics Methods

For each pellet, the three repeatedly acquired time-domain spectra were averaged and then transformed into the frequency-domain using the Fourier transform. The transmittance was defined as


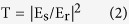


where E_s_ and E_r_ were the complex THz signals in the frequency-domain after fast Fourier transform (FFT) of the time-domain spectra of the sample and reference, respectively^36^. In this paper, derivative processes, including the first and second derivative, were selected as spectrum pretreatment methods^17^.

Chemometrics methods can highlight the chemical differences between samples and reduce the variation due to physical effects. PCA, DA and PLS methods have been proven to be effective in many applications^14^,^16^,^17^, and were therefore selected as the chemometrics methods in present study.

PCA was applied to obtain an overview of the data by extracting the main information from the THz spectra of the rice samples, reducing the number of variables and expressing the total variation in the data set in only a few PCs. Each spectrum will have its own unique set of scores, and therefore, a spectrum can be represented by its PCA scores in factor space instead of its intensities in wavelength space^28^. Because the PCs are orthogonal, by plotting the PCs, we can view the relationships between the different variables. Using either the pure spectral data or the pre-treated data, PCA can provide very important information about the potential capability of differentiating the samples^15^,^16^,^17^,^18^.

The DA method, which could be used to analyze samples with similar properties, is based on calculating the Mahalanobis distance of a sample from the centers of gravity of the considered groups^17^. If a sample is close to the center of gravity of its group, it is classified correctly. In the case where the distance to the center of gravity of another group is closer than that of its group, it is misclassified. In this work, the samples were divided into two groups: the transgenic group and the non-transgenic group.

PLS regression, a particular type of multivariate analysis that uses the two-block predictive PLS model to model the relationship between two matrices, could analyze data with strongly collinear (correlated), noisy, and numerous X-variables^18^. The PLS method could be appropriately used to enrich existing methodological approaches for strategic management research^37^.

In this study, chemometrics methods were employed to classify transgenic rice and their parents. The aim of this THz-TDS technique was to predict the membership of an individual to a qualitative group defined previously^38^.

All of the chemometrics calculations were performed using TQ Analyst V6.2.1 (Thermo Nicolet Corporation, Madison, WI, USA).

## Additional Information

**How to cite this article**: Xu, W. *et al.* Discrimination of Transgenic Rice containing the Cry1Ab Protein using Terahertz Spectroscopy and Chemometrics. *Sci. Rep.* **5**, 11115; doi: 10.1038/srep11115 (2015).

## Figures and Tables

**Table 1:** The partial least square results of the Cry1Ab pellets.

Data pretreatment	*r*_*c*_	*r*_*cv*_	RMSEC (%)	RMSECV (%)
no	0.9696	0.8824	0.87	1.73
1^st^ derivative	0.9725	0.8926	0.83	1.61
2^st^ derivative	0.9715	0.8912	0.84	1.63

**Table 2:** **The statistic results of the discriminating rice samples using discriminate analysis at different bands.**

**Range (THz)**	**Accuracy (%)**	**Range (THz)**	**Accuracy (%)**
0.1–0.2	72.4	0.1–0.8	84.0
0.1–0.3	80.4	0.1–0.9	84.7
0.1–0.4	77.3	0.1–1.0	85.3
0.1–0.5	83.4	0.1–1.1	85.3
0.1–0.6	85.3	0.1–1.2	85.3
0.1–0.7	84.0		

**Table 3:** The statistic results of THz spectra without and with derivative pretreatments by using discriminate analysis.

Data pretreatment	Numbers of misclassified transgenic rice and its parent in calibration set	Numbers of misclassified transgenic rice and its parent in validation set	Accuracy in calibration set (%)	Accuracy in validation set (%)
no	11, 7	4, 2	85.4	85.0
1^st^ derivative	9, 4	4, 2	89.4	85.0
2^st^ derivative	9, 4	4, 3	89.4	82.5
